# Identification of distinct and age‐dependent p16^High^ microglia subtypes

**DOI:** 10.1111/acel.13450

**Published:** 2021-10-01

**Authors:** Nynke Talma, Emma Gerrits, Boshi Wang, Bart J.L. Eggen, Marco Demaria

**Affiliations:** ^1^ Department of Biomedical Sciences of Cells & Systems Section Molecular Neurobiology University of Groningen University Medical Center Groningen Groningen The Netherlands; ^2^ European Research Institute for the Biology of Ageing University of Groningen University Medical Center Groningen Groningen The Netherlands

**Keywords:** Aging, cellular senescence, senescence, p16, neuroscience

## Abstract

Cells expressing high levels of the cyclin‐dependent kinase (CDK)4/6 inhibitor p16 (p16^High^) accumulate in aging tissues and promote multiple age‐related pathologies, including neurodegeneration. Here, we show that the number of p16^High^ cells is significantly increased in the central nervous system (CNS) of 2‐year‐old mice. Bulk RNAseq indicated that genes expressed by p16^High^ cells were associated with inflammation and phagocytosis. Single‐cell RNAseq of brain cells indicated p16^High^ cells were primarily microglia, and their accumulation was confirmed in brains of aged humans. Interestingly, we identified two distinct subpopulations of p16^High^ microglia in the mouse brain, with one being age‐associated and one present in young animals. Both p16^High^ clusters significantly differed from previously described disease‐associated microglia and expressed only a partial senescence signature. Taken together, our study provides evidence for the existence of two p16‐expressing microglia populations, one accumulating with age and another already present in youth that could positively and negatively contribute to brain homeostasis, function, and disease.

## Graphical abstract

P16+ microglia increases in aging brains of mice and humans. P16+ cells are found in 597 different microglia populations, including two previously unknown clusters.

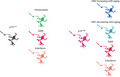

**Abbreviations**
AD: Alzheimer’s disease
CAM: CNS associated macrophages
CDK: Cyclin dependent kinase
CDP: Central animal facility
CNS: Central nervous system
DAM: Disease associated microglia
DEC: Animal care and use committee
GRP: Glial restricted progenitor
HOM: Homeostatic microglia
HSV: Herpes simplex virus
HVG: Highly variable feature
IFN: Interferon microglia
logFC: Log fold change
mRFP: Monomeric red fluorescent protein
MS: Multiple sclerosis
OPC: Oligodendrocyte progenitor cell
PCA: Principal component analysis
PD: Parkinson’s disease
qRT‐PCR: Quantitative real‐time poly chain reaction
SASP: Senescence associated secretory phenotype
tTK: thymidine kinase
UM: Unknown microglia
UMAP: Uniform manifold approximation and projection
WGCNA: Weighed gene correlation network analysis

## 1 INTRODUCTION

Cyclin‐dependent kinase (CDK)4/6 inhibitor p16^INK4a^ (from now on referred to as p16) levels gradually increase with age in multiple tissues and organisms (Herbig et al., 2006; Liu et al., 2009; Melk et al., 2004; Yousefzadeh et al., 2020). p16^High^ cells actively contribute to aging and age‐associated dysfunctions by restricting the regenerative potential of the tissue (Martin et al., 2014) and promoting chronic inflammation (Sanada et al., 2018). Genetic or pharmacological ablation of p16^High^ cells is able to increase health‐ and lifespan in mice (Baker et al., 2016; Xu et al., 2018). p16 expression is a common feature of cellular senescence (Liu et al., 2019), a state of stable and generally irreversible growth arrest originally described as a key process regulating cellular and organismal aging (Hayflick & Moorhead, 1961). Senescent cells are characterized by various structural changes, including misshaped nuclei, enhanced lysosomal content and phagocytic activity, altered mitochondria morphology, and changed plasma membrane composition (Hernandez‐Segura et al., 2018). In addition, senescent cells acquire a pro‐inflammatory phenotype by releasing cytokines and chemokines (a phenotype collectively defined as the SASP—senescence‐associated secretory phenotype) (Gorgoulis et al., 2019). Virtually, all cells can up‐regulate p16 levels, but this induction is not always reflected by a fully senescent state. For example, p16 expression is significantly increased in aged macrophages (Hall et al., 2016), but p16 overexpression can also be observed in young macrophages responding to physiological stimuli (Hall et al.,l., 2017), (Behmoaras & Gil, 2021).

Aging leads to a reduction in brain volume and cognition (Peters, 2006) and is the main risk factor for dementia and neurodegeneration (Wyss‐Coray, 2016). Aging and neurodegenerative conditions induce a common gene expression signature in microglia, the resident immune cells of the CNS (Galatro et al., 2017). Microglia exhibit a hypersensitive and pro‐inflammatory phenotype, known as priming, in particular during aging and neurodegeneration (Norden & Godbout, 2013; Perry & Holmes, 2014; Raj et al., 2014). These primed microglia exert an increased inflammatory response and thereby alter CNS function (Norden & Godbout, 2013). In addition to primed immune cells, the accumulation of pro‐inflammatory senescent cells in the CNS may also predispose elderly to neurodegenerative diseases or aggravate disease etiology (Kritsilis et al., 2018). In the CNS, p16 expression increases during natural aging and in brains affected by pathologies such as Parkinson's disease (PD), multiple sclerosis (MS), and Alzheimer's disease (AD) (Martin‐Ruiz et al., 2020; Nicaiseet al., 2019; Zhang et al., 2019). Removal of p16^High^ cells ameliorates the progression of neurodegeneration in amyloid and tau AD mouse models and in mice exposed to the neurotoxin paraquat (Bussian et al., 2018; Chinta et al., 2018; Zhang et al., 2019). In a neurodegenerative context, different cell types become p16^High^ and influence disease progression. A recent study has attempted to identify senescent cell types naturally occurring in the murine aging brain using single‐cell transcriptomic profiling, and identified an enrichment of p16^High^ cells in microglia and OPCs (Ogrodnik et al.,l., 2021). However, a limitation of single‐cell RNA sequencing (scRNAseq) is its ability to detect low abundant transcripts, which is the case of the *p16* transcript. Here, we aimed to identify p16^High^ cell populations in the aging brain by using a transgenic mouse model that allows for the isolation of cells expressing p16 at the protein level, and then perform validation of the findings in wild‐type mice and humans.

## 2 RESULTS

### 2.1 RFP^High^ cells expressing inflammatory and phagocytosis‐related genes accumulate in the aging brain of p16‐3MR mice

**FIGURE 1:**
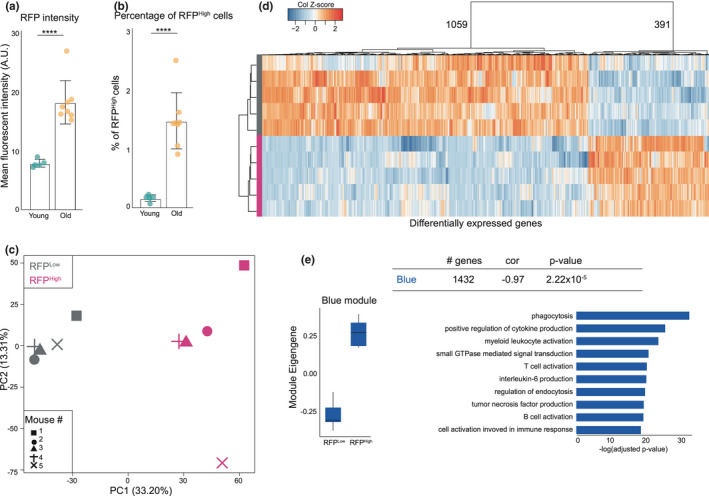
p16‐RFP expression is increased in the brain of aged p16‐3MR mice and abundantly express inflammatory and microglia genes. (a) Mean fluorescent RFP intensity of all viable cells in young compared to old brains. ****p<0,0001. (b) Percentage of viable cells positive for RFP in young mouse brains compared to old. ****p<0,0001. (c) PCA plot of bulk sequenced RFP^Low^ and RFP^High^ cells from old mouse brains. (d) Heatmap of all differentially expressed gene between the RFP^Low^ and RFP^High^ samples. E: Expression and gene‐ontology analysis of a WGCNA module enriched in RFP^High^ samples

The p16‐3MR mouse contains a monomeric red fluorescent protein (mRFP) fused to Renilla Luciferase and a truncated herpes simplex virus (HSV)‐1 thymidine kinase (tTK), under control of the p16 promoter (Demaria et al., 2014). In order to evaluate whether the levels of the 3MR transgene and the number of 3MR^High^ cells increase in the brain with age, we measured RFP signal and percentage of cells expressing high levels of RFP in 7‐ to 12‐week (defined young) and 105‐ to 116‐week (defined old) mice by flow cytometry (Figure S1a). The mean mRFP intensity was significantly higher in old mice (Figure 1a), and the percentage of cells expressing high levels of RFP (RFP^High^) cells increased >sevenfold with aging, from ~0.2% in young to ~1.5% in old mouse brains (Figure 1b). Importantly, the purified RFP^High^ population was enriched in cells expressing high levels of the p16 transcript (Figure S1b).

We then isolated RFP^Low^ and RFP^High^ cells from aged brains and generated gene expression profiles of both populations using bulk RNA sequencing (RNAseq). Principal component analysis (PCA) showed significant transcriptional differences between the RFP^Low^ and RFP^High^ populations as indicated by the first principal component (Figure 1c). Differential gene expression analysis revealed 1459 differentially expressed genes between the two populations (Figure 1d). Among the most enriched genes in the RFP^High^ samples (Table S1) were *Cass4* and *Apba2* (or *Mint2*), which are involved in amyloid synthesis and AD (Beck et al., 2014; Ho et al., 2008) and genes associated with macrophage activation, like *Akr1b3*, *Angptl7*, and *Ticam2* (Qian et al., 2016; Ramana et al., 2006; Seya et al., 2005).

To determine whether gene networks in RFP^High^ samples associated with specific biological or cellular functions, a weighted gene correlation network analysis (WGCNA) (Langfelder & Horvath, 2008) was performed, resulting in branches, or modules, of highly correlating genes (Figure S1c; Table S2). One of these modules (the “blue” module), involved in phagocytosis and cytokine production, was significantly enriched in the RFP^High^ samples, as reflected by the Module Eigengene, or first principal component, of the module (Figure 1e; Figure S1d‐h). These data suggest that RFP^High^ cells accumulate in the aging brain and are enriched in expression of genes associated with inflammation and phagocytosis pathways.

### 2.2 Single‐cell transcriptomic profiling demonstrates accumulation of RFP^High^ microglia with aging in p16‐3MR mice

**FIGURE 2:**
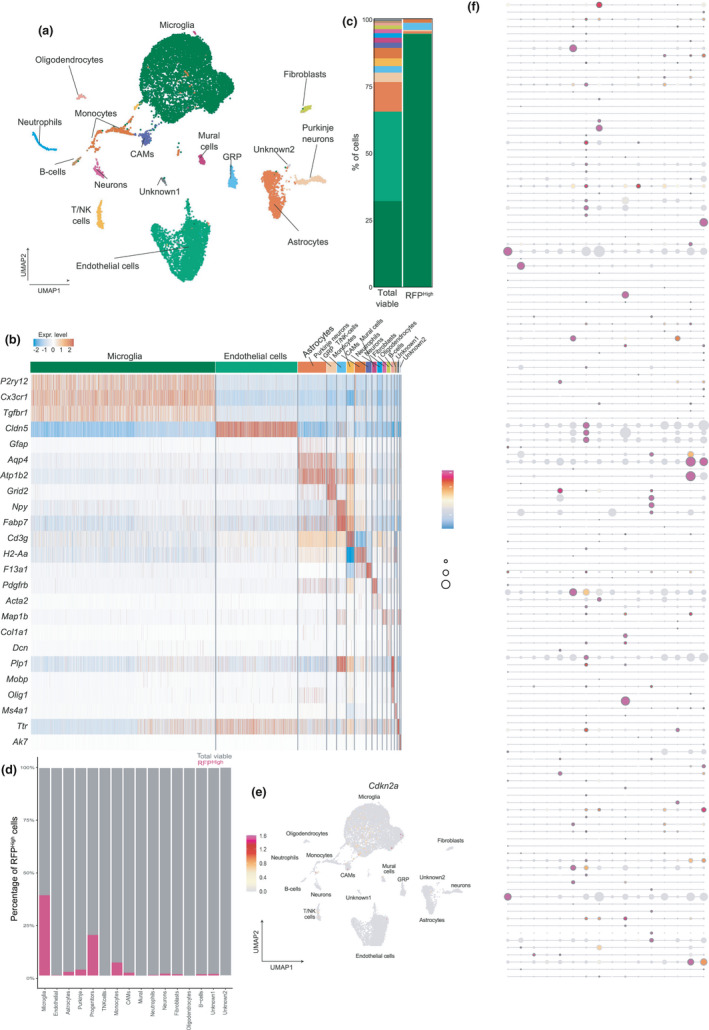
RFP^High^ cells are highly enriched for microglia. (a) UMAP depicting mouse CNS with cluster annotations based on cell types. (b) Heatmap showing the expression of cell type markers in each cluster. (c) Barplot of cluster distribution of total viable cells and RFP^High^ cells. (d) Barplot showing the percentage of RFP^High^ cells for each cell type. (e) *Cdkn2a* plotted in UMAP of all sequenced single cells. (f) Dotplot showing the expression of senescence markers in each cluster

To further characterize the phenotype of the RFP^High^ cell population in the aged mouse CNS, we compared scRNAseq profiles of purified RFP^High^ cells to unsorted CNS cell samples (Figure S2a‐d; Table S3). We identified 14 clusters in the dataset, using unsupervised, graph‐based clustering analysis where each cluster corresponds to a distinct cell type (Figure 2a). The cell types were identified based on the expression of well‐known cell type marker genes: *P2ry12*, *Cx3cr1*, and *Tgfbr1* for microglia; *Cldn5* for endothelial cells; *Gfap*, *Aqp4*, and *Atp1b2* for astrocytes; *Grid2* for Purkinje neurons; *Npy* and *Fabp7* for glial restricted progenitors (GRP); *Cd3g* for T/NK cells; *H2*‐*Aa* for monocytes; *F13a1* for CNS‐associated macrophages (CAMs); *Pdgfrb* for mural cells; *Acta2* for neutrophils; *Map1b* for neurons; *Dcn* and *Col1a1* for fibroblasts; *Olig1*, *Mobp*, and *Plp1* for oligodendrocytes; *Ms4a1* for B cells; *Ttr* for unidentified population 1 (unknown 1); and *Ak7* for unidentified population 2 (unknown 2) (Figure 2b; Table S4). Next, for the total viable and the RFP^High^ populations, the distribution of cell types within each sample was compared. Microglia, astrocytes, and endothelial cells were the most abundant cell types obtained with our isolation method (total viable population) from aged mouse brains, while other cell types such as neurons and oligodendrocytes were less abundant, and most likely underrepresented compared to their normal physiological distribution in the CNS (Valério‐Gomes et al., 2018). Strikingly, the RFP^High^ sample was almost exclusively comprised of microglia (94.6%) and some glial restricted progenitors (2.6%) (Figure 2c and d).

The scRNAseq data confirmed that microglia expressed *Cdkn2a*, the genomic locus containing *p16*, more abundantly compared to other cell types in the CNS (Figure 2e). To investigate whether microglia showed additional markers of cellular senescence, the expression levels of a list of 162 senescence‐associated genes in each cell type were evaluated (Table S5). These genes were variably expressed and not abundantly present in the microglia population (Figure 2f). These data suggest that RFP^High^ microglia accumulate in the aging brain of p16‐3MR mice and that their transcriptional profile differs from a classical senescence‐associated gene signature.

### 2.3 Microglia are enriched in *p16* in the brains of wild‐type mice and humans

**FIGURE 3:**
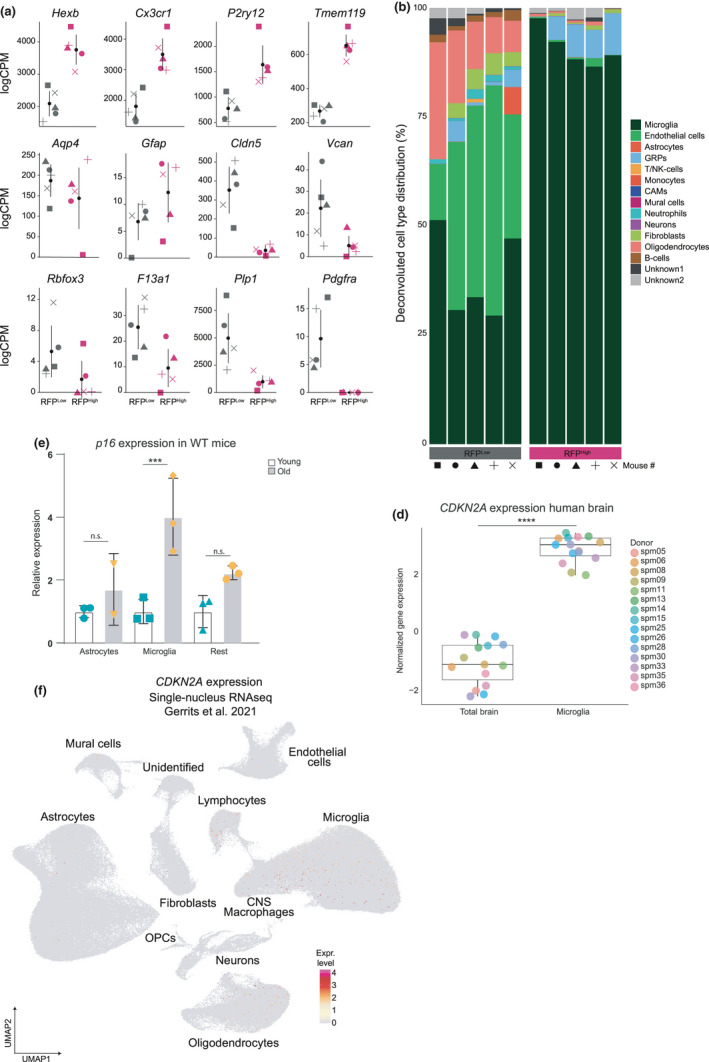
Increased expression of p16 in mouse and human microglia. (a) Gene expression of cell marker genes in RFP^Low^ compared to RFP^High^ mouse samples. (b) Barplot showing the distribution of cells types in the mouse CNS bulk dataset after deconvolution. (c) *p16* expression measured by qPCR in cells isolated from young and old mouse brains. ****p<0,0001. (d) *CCKN2A* expression in human microglia and total cortical tissue (from Galatro et al., 2017). ****p<0,0001E: UMAP depicting *CDKN2A* expression in 450,000 CNS cell nuclei (Gerrits et al. 2021)

To confirm the presence of RFP^High^ microglia in aged brains, we used different methods. First, from the bulk RNAseq list, we investigated the expression level of cell type‐specific genes in the RFP^High^ fraction: *Hexb*, *Cxcr1*, *P2ry12*, and *Tmem119* for microglia; *Aqp4* and *Gfap* for astrocytes; *Cldn5* and *Vcan* for endothelial cells; *Rbfox3* for neurons; *F13a1* for CNS‐associated macrophages; *Plp1* for oligodendrocytes; and *Pdgfra* for oligodendrocyte progenitor cells and fibroblasts (Figure 3a). The expression level of microglia genes was consistently higher in the RFP^High^ samples, while in the RFP^Low^ samples, endothelial cell, oligodendrocyte, and oligodendrocyte progenitor cell markers were more abundantly expressed. Second, we deconvoluted transcriptomes of the bulk RFP^High^ samples with CIBERSORT, using our single‐cell data as the reference matrix (Table S4). Again, a pattern of enrichment for microglia in the RFP^High^ cell population was observed (Figure 3b).

To validate the correlation between p16 and RFP positivity in a non‐transgenic background, we measured p16 levels in wild‐type animals. We isolated microglia, astrocytes, and non‐microglia/non‐astrocyte (defined as “the rest”) cells from the brain of young and old wild‐type C57BL/6 mouse brains and evaluated the *p16* transcript levels of the isolated populations. Only microglia of old mice revealed a significant *p16* upregulation, while no significant differences between young and old mice were detected neither in astrocytes, a cell population that was minimally represented in the RFP^High^ cells isolated from aged p16‐3MR mice, nor in other mixed cell types mainly consisting of endothelial cells (Figure 3c).

Next, we evaluated the level of *p16* expression in human microglia and cortical CNS tissue (Galatro et al., 2017). Strikingly, we measured a significant enrichment for *CDKN2A*, the genomic locus containing *p16*, in the microglia population compared to the total brain samples (Figure 3d). In addition, we determined the expression levels of *CDKN2A* in a single‐nucleus RNA sequencing data set of human AD cases and healthy donors (Gerrits et al., 2021). Also in this dataset, *CDKN2A* was most abundantly expressed by microglia (Figure 3e). Interestingly, lymphocytes and oligodendrocytes, underrepresented in our mouse scRNAseq, also expressed *CDKN2A* in human brains. Altogether, these data confirm that both in the mouse and in the human aged brain, p16^High^ cells are mostly present in the microglia population.

### 2.4 RFP^High^ cells cluster in two distinct and previously unreported microglia populations

**FIGURE 4:**
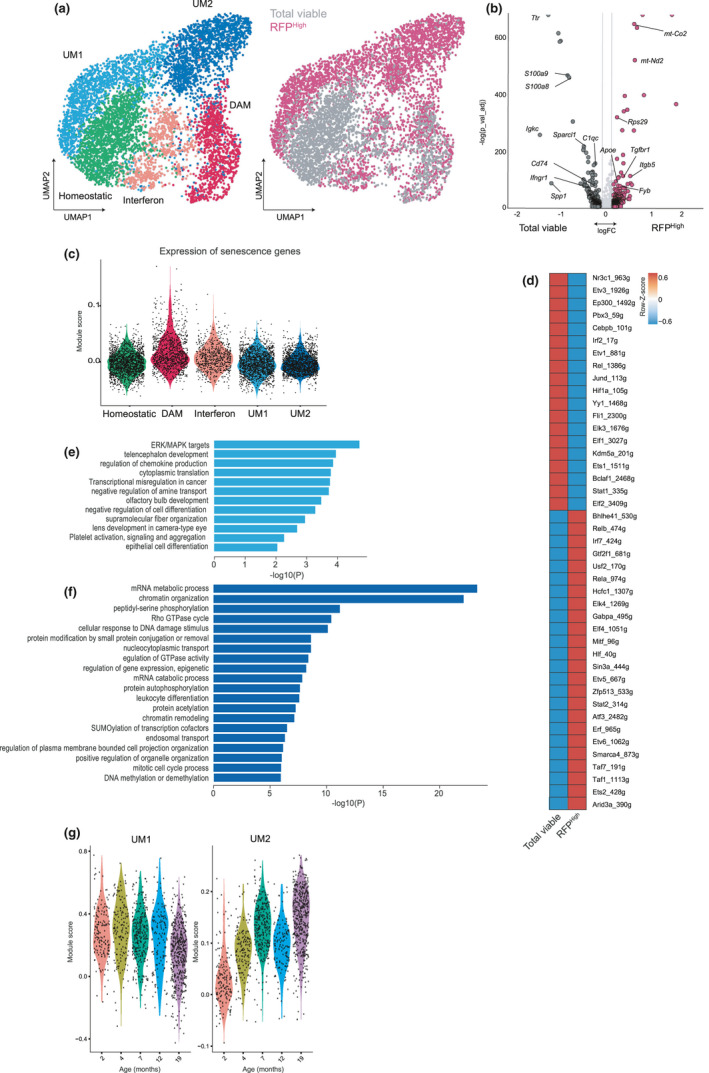
p16^High^ microglia express genes associated with inflammation, cell cycle response, and cell motility. (a) UMAP plots where colors indicate the different clusters within all the sequenced microglia cells. DAM=damage‐associated microglia. (b) Volcano plot depicting differential expressed genes between the RFP^High^ microglia and total viable microglia. (c) Violin plot showing the expression of senescence genes in each microglia cluster. (d) Heatmap showing the differentially expressed regulons in the SCENIC analysis between all RFP^High^ and total viable microglia. (e) GOs significantly enriched in the p16‐UM1 cluster. (f) GOs significantly enriched in the p16‐UM2 cluster. (g) Violin plot depicting the expression of UM1 and UM2 cluster markers with age in wild‐type mice of the dataset from Zhang et al. (Zhang et al., 2020)

Recent reports based on single‐cell transcriptomes identified context‐dependent microglia subtypes (Masuda et al., 2020; Sierksma et al., 2020). Subclustering analysis of the entire microglia population from our single‐cell dataset (RFP^High^ and unpurified) revealed 5 distinct subpopulations: 3 previously described—a population which surveils the surroundings and maintains homeostasis through clearance of cellular debris, called homeostatic (HOM); a more reactive population, which acquires pro‐inflammatory and antigen‐presenting properties, called disease‐associated microglia (DAM); and activated microglia with high interferon signaling (IFN)—and 2 additional clusters, named unknown microglia clusters 1 and 2 (UM1 and UM2), which segregated from the known clusters and were almost exclusively derived from the RFP^High^ samples (Figure 4a; Figure S3a). The HOM cluster was depleted in the RFP^High^ microglia, while DAM and IFN clusters were equally present in both RFP^High^ and RFP^Low^ populations. Differential gene expression analysis revealed a clear distinction of the RFP^High^ microglia from the total viable population (Figure 4b), even if the expression of selected senescence‐associated genes was not specifically enriched in the UM1 and UM2 clusters, but seems to be slightly increased in the DAM cluster (Figure 4c; Figure S3d). Single‐cell regulatory network inference and clustering (SCENIC) analysis identified 43 gene networks differentially expressed between RFP^High^ and total microglia. Interestingly, expression of genes regulated by *Ets2*, a transcription factor that positively regulates p16 expression (Kotake et al., 2015), was enriched in RFP^High^ microglia (Figure 4d; Figure S3b).

We then investigated the predicted functions of genes upregulated in the RFP^High^ microglia. In line with our bulk RNAseq results, two AD risk genes were upregulated in the RFP^High^ microglia. *Gsap* selectively increases amyloid‐beta production (He et al., 2010), a protein that is aggregated in AD and inositol polyphosphate‐5‐phosphatase D (*Inpp5d)* is suggested to contribute to AD in a non‐amyloid‐beta‐dependent fashion (Efthymiou & Goate, 2017). Additionally, we found genes involved in macrophage motility and myelination. *Plxnb2* has been shown to negatively regulate cell motility (Roney et al., 2011), while *Kif13b* regulates myelination in the CNS (Noseda et al., 2016) (Table S4). In addition, we examined the genes upregulated in each UM cluster. Gene ontology analysis for genes enriched in the UM1 cluster showed an enrichment for genes involved in the ERK/MAPK pathways (Figure 4e) suggested to underlie CNS inflammation (Kaminska et al., 2009). Genes highly expressed in UM2 microglia were associated with cell cycle response and Rho GTPase signaling (Figure 4f), a pathway necessary for process motility, which is important for scanning of the parenchyma (Neubrand et al., 2014).

Finally, we compared the gene expression profile of the RFP^High^ microglia to previously reported disease‐ and aging‐associated microglia profiles (Table S5). While both the DAM and the IFN clusters significantly overlap with previously reported profiles, none of the investigated gene sets was significantly enriched in our UM1 and UM2 clusters (Figure S3c). Interestingly, when we looked at the expression levels of UM1 and UM2 cluster marker genes in aging wild‐type mice from the dataset of Zhang et al. 2020, we observed that UM1 cluster markers were expressed in microglia at all ages albeit lower at 19 months, while the expression of UM2 cluster marker genes progressively increased with age in these wild‐type mice (Figure 4g). In summary, these data show that RFP^High^ microglia cluster in two distinct subpopulations with previously unreported gene signatures which we named UM1 and UM2. UM1 negatively correlates with age and is characterized by expression of inflammatory genes. In contrast, UM2 is age‐associated and characterized by differential expression of genes involved in cell cycle regulation and cell motility.

## 3 DISCUSSION

Microglia, tissue‐resident macrophages of the CNS, is a heterogeneous cell population that change over the course of an organism lifespan. Microglia heterogeneity decreases with age, but several states—for example chemokine‐enriched inflammatory microglia—remain unchanged or increase in aged brains (Hammond et al., 2019). Moreover, microglia are reported to age in a regional‐dependent manner (Grabert et al., 2016). However, there is still little understanding of the phenotypical characteristics of microglia subpopulations in the aged brain. The current study reveals two previously unreported p16‐expressing microglia subpopulations, one with a quite stable expression across different life stages and one which accumulation significantly increases with age.

Elevated p16 expression is a marker of cellular senescence and has been used to identify the accumulation of senescent astrocytes (Bhat et al., 2012; Chinta et al., 2018; Yabluchanskiy et al., 2020), oligodendrocyte progenitor cells (Nicaise et al., 2019; Zhang et al., 2019), and neurons in the human aging brain (Kang et al., 2015) and in mouse models of neurodegeneration. Moreover, recent data indicated that microglia accumulate p16^High^ cells in aged mouse brains (Ogrodnik et al., 2021).

In this study, using both transgenic and wild‐type mice, and various publicly available mouse and human transcriptomic datasets, we identified two distinct subpopulations of p16^High^ microglia, one constantly present and one age‐associated, that did not express a classical senescence‐associated gen signature. Absence of a senescence profiling is in line with a previous study showing that while murine microglia *in vitro* show markers of replicative senescence, the microglia of aged mice express higher levels of p16 but not other typical senescence‐associated changes (Stojiljkovic et al., 2019).

Distinct transcriptional changes in each cell population were found during single‐cell sequencing of the aged murine brain (Ximerakis et al., 2019), indicating that each cell type ages differently. In our single‐cell study, only astrocytes, endothelial cells, and microglia were represented in large quantities, while other cell types were underrepresented due to our cold protease isolation procedure. Since we also identified higher expression of *CDKN2A* in lymphocytes and oligodendrocytes by analyzing a dataset derived from RNAseq of single nuclei isolated from human brains (Gerrits et al., 2021), it remains to be seen whether other less represented populations also express p16 with age.

Our data suggest a clear separation of the p16^High^ microglia from other microglia populations and the existence of two distinct subsets—one expressed across the entire lifespan and the other age‐associated. A subset of p16^High^ microglia may be part of a homeostatic mechanism aimed at reducing damage propagation, via cell cycle arrest and improved phagocytic properties, and at promoting immune surveillance, via activation of specific secretory and pro‐inflammatory phenotypes. On the other side, the accumulation of a subset of p16^High^ cells with age may represent the byproduct of excessive damage and reduced clearance capacity, which could contribute to detriment accumulation and loss of tissue homeostasis. Future studies need to address this issue by evaluating the effects of specifically eliminating specific p16^High^ microglia subsets, and to further characterize the presence and function of these subsets in the human brain. It will also be important to evaluate whether current senolytic approaches are eliminating these p16^High^ microglia subsets, and the balance between benefits and toxicities of removing such populations.

## 4 MATERIALS AND METHODS

### 4.1 Mice

p16–3MR mice with a C57BL/6 background or wild‐type C57BL/6 were used for all experiments (Demaria et al., 2014). Young mice were between 7 and 12 weeks of age, and old mice were between 105 and 116 weeks of age. The young mice were a mix of males and females (n=5), male old mice were used for bulk sequencing (n=5), and female mice were used for single‐cell sequencing (n=4). Young, 18 weeks of age, (n=3) and old, 101 and 104 weeks of age, (n=3) wild‐type mice were used for the isolation of astrocytes, microglia, and rest cells. Mice were raised on a 12‐hr light/dark cycle with food and water available *ad libitum* and were individually housed. All experiments were performed in the Central Animal Facility (CDP) of the UMCG, with protocol (15339–02–001) approved by the Animal Care and Use Committee (DEC) of the University of Groningen.

### 4.2 Cell isolation from mouse brain tissue

Cells were isolated from adult mouse brain using an enzymatic protocol at 4℃. The brains were isolated and dissociated by three rounds of GentleMACS (m_brain_01, m_brain_02, and m_brain_03) in enzyme mix of 15 mg/ml Protease (Sigma P5380), 1 mM L‐cysteine hydrochloride (Sigma C7477), and 0.5 µg/µl DNase (Roche 10104159001) with 10 min incubation in the mix on ice in between GentleMACS programs. The homogenized brain samples were passed through a 100 μM cell strainer to obtain a single‐cell suspension. The cells were centrifuged at 300 rcf for 10 min at 4℃, and the pellet was resuspended in 24% Percoll gradient buffer. 3 mL dPBS was pipetted onto the gradient buffer, and myelin was removed by centrifuging at 950 rcf for 20 min at 4℃. The cell pellets were incubated with DAPI and Draq5. Viable cells were FACS sorted as DAPI^neg^Draq5^pos^ events. RFP^High^ and RFP^Low^ bulk samples were sorted from individual mice, but for the single‐cell sequencing, RFP^High^ (21,500) and total viable cells (45,000) from four mice were combined each into one lane of a 10X Genomics Chromium chip.

For the isolation of astrocytes, microglia, and rest cells, cell pellets were incubated with the antibodies CD11b‐BV421 (clone M1/70, Biolegend, San Diego, CA, USA), CD45‐FITC (clone 30‐F11, Biolegend, San Diego, CA, USA), CD49d‐PE (clone R1‐2, Miltenyi Biotec), Acsa2‐FITC (clone REA969, Miltenyi Biotec), PI, and Draq5. Microglia were FACS sorted as PI^neg^ Draq5^pos^ CD11b^high^ CD45^int^ CD49d^neg^ events. Astrocytes were FACS sorted as PI^neg^ Draq5^pos^ CD11b^neg^ CD45^neg^ Acsa2^pos^ events and rest cells as PI^neg^ Draq5^pos^ CD11b^neg^ CD45^neg^ Acsa2^neg^ events. Bulk samples were sorted from individual mice.

### 4.3 FACS analysis

Flowjo V.10 was used to analyze the mean, median RFP expression, number of RFP positive cells, and viability of cells. Unpaired t tests were used to compare the mean, median, and number of positive cells. Paired t test was used to compare viability.

### 4.4 Real‐Time PCR

Total RNA was prepared using the AllPrep DNA/RNA Micro Kit (Qiagen, 80284). RNA was reverse transcribed into cDNA using a kit (Applied Biosystems). Quantitative RT‐PCR (qRT‐PCR) reactions were performed as described (Demaria et al., 2010) using the Universal Probe Library system (Roche). Primer used:

mp16 #91 ‐FAATCTCCGCGAGGAAAGC ‐RGTCTGCAGCGGACTCCAT.

mHprt1 #62 ‐FATCACATTGTGGCCCTCTG ‐RGTCATGGGAATGGATCTATCACT.

mHmbs #91 ‐FAGAAAAGTGCCGTGGGAAC ‐RTGTTGAGGTTTCCCCGAAT.

### 4.5 Bulk RNAseq library construction and sequencing

RNA was isolated from cell pellets with the AllPrep DNA/RNA Micro Kit (Qiagen, 80284). RNA concentrations were measured on a Qubit using a HS RNA kit. 2,5 ng of the samples was used for library preparation with the Lexogen QuantSeq 3’ mRNA‐Seq Library Prep Kit (FWD) from Illumina. All libraries were pooled equimolarly and sequenced on a NextSeq 500 at the sequencing facility in the UMCG.

### 4.6 scRNAseq library construction and sequencing

The single‐cell cDNA libraries were constructed using the Chromium Single Cell 3’ Reagents Kit v3 and corresponding user guide (10x Genomics). All samples were pooled in equimolar ratios and sequenced on a NextSeq 500 at the sequencing facility in the UMCG.

### 4.7 Gene sets from literature

To compare our microglia clusters with reported microglia phenotypes in literature, several gene sets were downloaded. From (Sierksma et al., 2020), EV7 was downloaded and genes with a p_val_adj <0.05 and logFC >0.15 were selected (304 genes) and from EV6 the CPM gene set (521 genes). From (Hammond et al., 2019), table S1 was downloaded and marker genes from clusters OA2 and OA3 were selected (136 and 37 genes, respectively). From (Keren‐Shaul et al., 2017), table S2 was downloaded and upregulated genes of “Microglia3” with a p_val_adj <0.05 were selected (469 genes). From (Butovsky & Weiner, 2018), upregulated genes listed in Figure 2 were used (29 genes). From (Gerrits et al., 2020), genes from table S4 with a p_val_adj <0.05 and logFC >0.15 were selected (188 genes). From Galatro et al. (2017), Voom Normalized counts were downloaded from GEO. From Gerrits et al. 2021, the exact same analyzed data objects as reported in the paper were used as these were generated by ourselves.

### 4.8 Bulk RNAseq data analysis

Data preprocessing was performed with the Lexogen Quantseq 2.3.1 FWD UMI pipeline on the BlueBee Genomics Platform (1.10.18). Count files were loaded into R, and DAFS filtering was performed to remove lowly expressed genes (George & Chang, 2014). A negative binomial generalized log‐linear model was used to model gene expression levels, as implemented in edgeR, adjusted for mouse since the RFP^Low^ and RFP^High^ cells were obtained from the same mice and differentially expressed genes were determined using a likelihood ratio test (Robinson et al., 2010). Thresholds were set at abs(logFC) >1 and *p* < 0.05. Principal component analysis was performed on logCPM transformed counts. Visualizations were made with the CRAN package “ggplot2.” Heatmaps were made with the CRAN package “gplots,” and rows and columns were clustered using hierarchical clustering with the ward.D2 method on Pearson's correlations. For WGCNA analysis, VST‐transformed counts obtained from DESeq2 were used as input (Langfelder & Horvath, 2008; Love et al., 2014). Signed WGCNA was performed using biweight mid‐correlations, and the max number of excluded outliers was restricted to 10%. Since we were dealing with binary data (i.e., two experimental groups), the robust treatment for the y variable of the biweight mid‐correlation was turned off (Langfelder & Horvath, 2012). Gene ontology analysis was performed on significantly differentially expressed genes (*p* < 0.05 and logFC >0.15) using “clusterProfiler” with a p‐ and q‐value cutoff of 0.05.

### 4.9 scRNAseq data analysis

Raw reads were processed using Cell Ranger 3.0.0 with default settings and aligned to the mouse mm10 genome. Barcode filtering was performed with DropletUtils with a threshold on >250 UMIs. Counts from cellular barcodes were then extracted from the raw output count matrix from Cell ranger. Cells with a mitochondrial content >10% were removed from the dataset. Counts from the different sample groups were merged into one using the “Merge” function from Seurat (v3). Then, the data were SCTransformed with regression on mitochondrial and ribosomal content, and subsequently, PCA, UMAP, finding neighbors, and clustering were performed as implemented by Seurat (Hafemeister & Satija, 2019). For differential gene expression analysis, raw counts were normalized using the “NormalizeData” function; then, DE genes were identified with MAST. Geneset scores were calculated using the “AddModuleScore” function. Average gene expression per cluster was calculated using the “AverageExpression” function. Median of expressed genes that were mitochondrial per cell: 2.2%; ribosomal: 5.6%; and median number of genes detected per cell: 755.

Regulatory gene network (regulon) analysis was performed using SCENIC; normalized counts from Seurat were used as input (Aibar et al., 2017). Only genes with more than 3 counts and present in at least 0.5% of all cells were included. GENIE3 and SCENIC were used with default settings (Huynh‐Thu et al., 2010; Aibar et al., 2017). Enrichment of gene sets and regulons in our scRNAseq data was quantified using AUCell. AUC values are plotted as an average per group. Regulons with a median AUC <0.01 were excluded in the downstream analysis.

From Zhang et al. (2020), the raw count matrices of all mice were downloaded and raw reads were processed using Cell Ranger 3.0.0 with default settings and the pre‐mRNA package. From the bam file, exonic reads and intronic reads mapping in the same direction as the mRNA were counted per barcode with Abacus in order to distinguish barcodes containing nuclear RNA from ambient and cytoplasmic RNA (Xi et al., 2020). The counts corresponding to these barcodes were extracted from the raw count matrix generated by Cell Ranger and loaded in R with Seurat (3.0.3). Nuclei with a mitochondrial content >5% were removed from the dataset. Count matrices of all mice were merged. The data were normalized for library size, by a scale factor of 10,000 and log‐transformed. Scrublet was used to identify and remove doublets (Wolock et al., 2019) (Wolock et al., 2019). Highly variable features (HVGs) were determined using the VST method. The data were scaled and heterogeneity associated with number of UMIs and mitochondrial content was regressed out and the data were clustered using the graph‐based clustering approach implemented in Seurat. The microglia cluster was identified based on expression of *P2ry12*, *Csf1r*, and *Cx3cr1*. Then, only WT mice were used for further analysis. Geneset scores were calculated using the “AddModuleScore” function from Seurat.

## CONFLICT OF INTEREST

MD is co‐founder, shareholder, and advisor for Cleara Biotech. The project was not funded or influenced by Cleara.

## AUTHOR CONTRIBUTIONS

N.T., E.G., and B.W. involved in methodology. N.T. and E.G. involved in validation and formal analysis. N.T., E.G., B.W., B.E., and M.D. involved in investigation. N.T., B.E., and M.D. involved in writing–original draft preparation, conceptualization, and writing–review and editing. B.E. and M.D. involved in supervision and funding acquisition.

## Supporting information

Figure S1‐S3

Table S1‐S7

## Data Availability

RNAseq data are deposited in the database GEO (www.ncbi.nih.gov/geo/) with identifier GSE151459. All the data presented here are available from the corresponding authors upon reasonable request.
