# Supplementary material for: Identification of distinct and age‐dependent p16High microglia subtypes
Source: Aging Cell. 2021 Oct 1;20(10):e13450. doi: 10.1111/acel.13450 (PMC8520715; doi:10.1111/acel.13450)
Supplement: Supplementary file 1 — Figure S1‐S3 [file ACEL-20-e13450-s002.pdf]

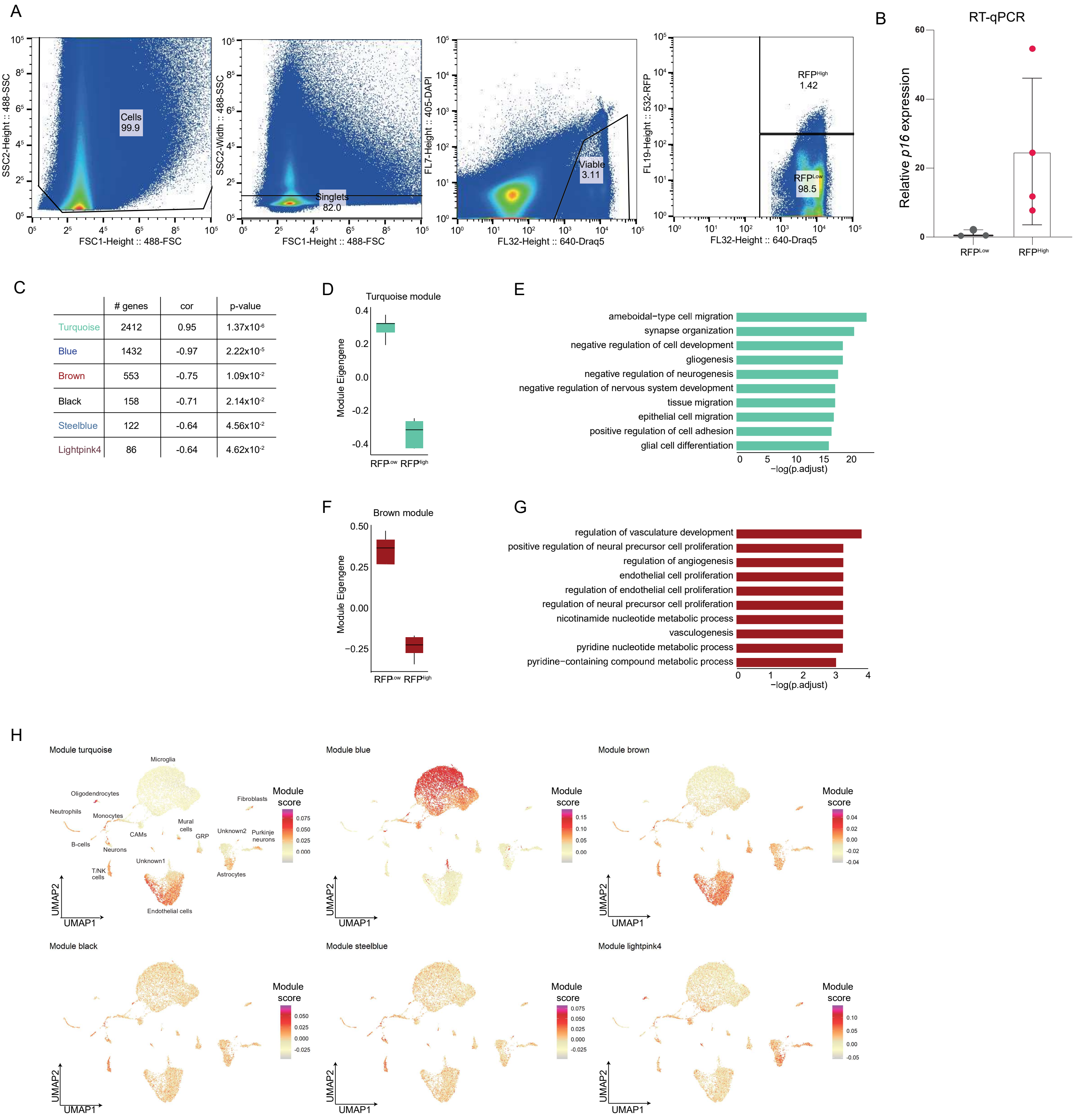

	# genes	cor	p-value
Turquoise	2412	0.95	$1.37x10^{-6}$
Blue	1432	-0.97	$2.22x10^{-5}$
Brown	553	-0.75	$1.09x10^{-2}$
Black	158	-0.71	$2.14x10^{-2}$
Steelblue	122	-0.64	$4.56x10^{-2}$
Lightpink4	86	-0.64	$4.62x10^{-2}$

Figure S1: WGCNA module expression between cell types.

A: Gating strategy for sorting RFP^{High} cells by FACS. B: p16 expression measured by qPCR in old mouse brains. C: List of significant weighted gene correlation network analysis (WGCNA) modules between RFP^{Low} and RFP^{High} samples. D-G: ModuleEigengene expression levels and gene ontology analysis of WGCNA modules enriched in RFP^{Low} samples. H: UMAPs depicting the expression of WGCNA modules in all isolated cell types in single cell data set.

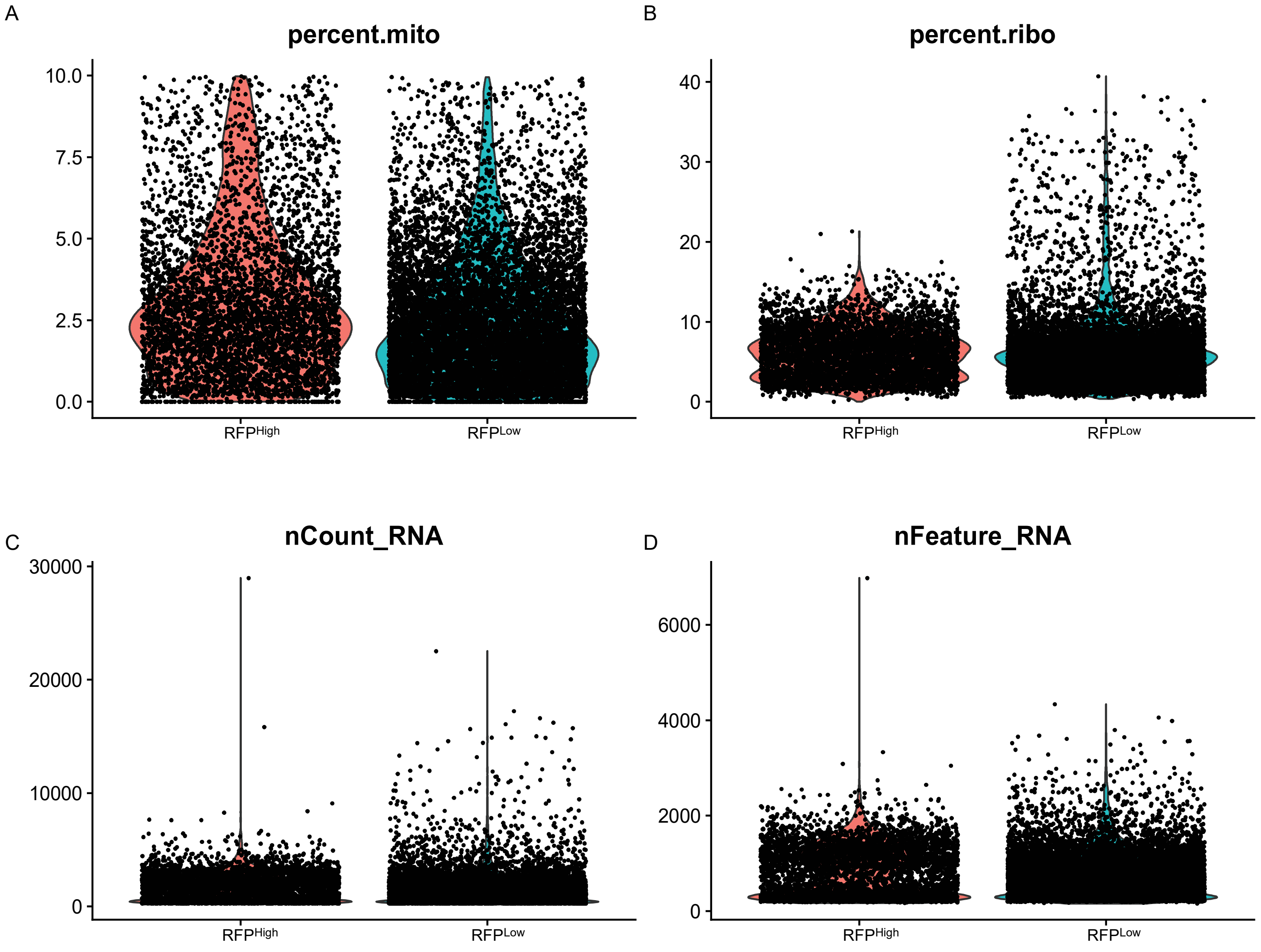

Figure S2: Quality control of scRNAseq

A: Percentage mitochondrial RNA, B: percentage ribosomal RNA, C: read counts and D: number of unique genes detected in RFP^{Low} and RFP^{High} samples.

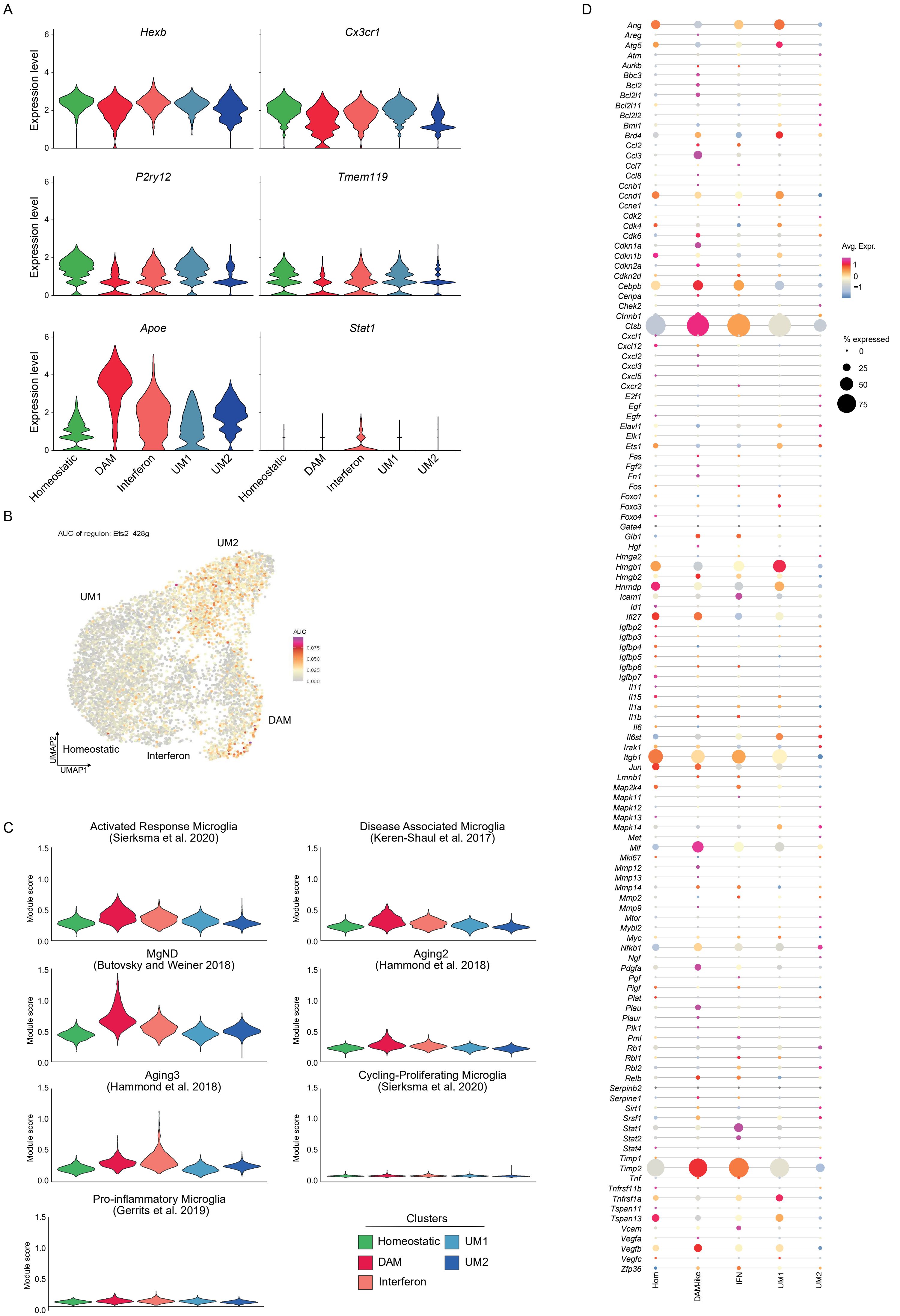
A
Hexb
Cx3cr1
P2ry12
Tmem119
Apoe
Stat1
Expression level
Homeostatic
DAM
Interferon
UM1
UM2
B
AUC of regulon: Ets2_428g
UM2
UM1
DAM
Homeostatic
Interferon
UMAP2
UMAP1
AUC
0.075
0.050
0.025
0.000
C
Activated Response Microglia
(Sierksma et al. 2020)
Disease Associated Microglia
(Keren-Shaul et al. 2017)
MgND
(Butovsky and Weiner 2018)
Aging2
(Hammond et al. 2018)
Aging3
(Hammond et al. 2018)
Cycling-Proliferating Microglia
(Sierksma et al. 2020)
Pro-inflammatory Microglia
(Gerrits et al. 2019)
Module score
Clusters
Homeostatic
UM1
DAM
UM2
Interferon
D
Ang
Areg
Atg5
Atm
Aurkb
Bbc3
Bcl2
Bcl2l1
Bcl2l11
Bcl2l2
Bmi1
Brd4
Ccl2
Ccl3
Ccl7
Ccl8
Ccnb1
Ccnd1
Ccne1
Cdk2
Cdk4
Cdk6
Cdkn1a
Cdkn1b
Cdkn2a
Cdkn2d
Cebpb
Cenpa
Chek2
Ctnnb1
Ctsb
Cxcl1
Cxcl12
Cxcl2
Cxcl3
Cxcl5
Cxcr2
E2f1
Egf
Egfr
Elavl1
Elk1
Ets1
Fas
Fgf2
Fn1
Fos
Foxo1
Foxo3
Foxo4
Gata4
Glb1
Hgf
Hmga2
Hmgb1
Hmgb2
Hnrndp
Icam1
Id1
Ifi27
Igfbp2
Igfbp3
Igfbp4
Igfbp5
Igfbp6
Igfbp7
Il11
Il15
Il1a
Il1b
Il6
Il6st
Irak1
Itgb1
Jun
Lmnb1
Map2k4
Mapk11
Mapk12
Mapk13
Mapk14
Met
Mif
Mki67
Mmp12
Mmp13
Mmp14
Mmp2
Mmp9
Mtor
Mybl2
Myc
Nfkb1
Ngf
Pdgfa
Pgf
Pigf
Plat
Plau
Plaur
Plk1
Pml
Rb1
Rbl1
Rbl2
Relb
Serpinb2
Serpine1
Sirt1
Srsf1
Stat1
Stat2
Stat4
Timp1
Timp2
Tnf
Tnfrsf11b
Tnfrsf1a
Tspan11
Tspan13
Vcam
Vegfa
Vegfb
Vegfc
Zfp36
Hom
DAM-like
IFN
UM1
UM2
Avg. Expr.
1
0
−1
% expressed
0
25
50
75

Figure S3: Microglia clusters in literatue do not resemble p16^{pos} microglia.

A: Violin plot depicting the expression of Hexb, Cxcr1, P2ry12,Tmem119, Apoe and Stat1 respectively in each cell of the microglia cluster. B: UMAP depicting Ets2 regulon expression in all microglia. C: Violin plots showing the expression of subtype signatures from the literature in each cluster. D:Dot plot showing expression of senescence genes in microglia clusters.
